# The Living, Dynamic and Complex Environment Care in Intensive Care Unit^1^

**DOI:** 10.1590/0104-1169.0568.2570

**Published:** 2015-07-03

**Authors:** Marli Terezinha Stein Backes, Alacoque Lorenzini Erdmann, Andreas Büscher

**Affiliations:** 2 PhD, Adjunct Professor, Departamento de Enfermagem, Universidade Federal de Santa Catarina, Florianópolis, SC, Brazil; 3 PhD, Full Professor, Departamento de Enfermagem, Universidade Federal de Santa Catarina, Florianópolis, SC, Brazil; 4 PhD, Professor, Hochschule Osnabrück, Fakultät Wirtschafts und Sozialwissenschaften, Osnabrück, Nothwesfallen, Germany

**Keywords:** Systemic Management, Health Facility Environment, Intensive Care Units

## Abstract

**OBJECTIVE:**

to understand the meaning of the Adult Intensive Care Unit environment of care, experienced by professionals working in this unit, managers, patients, families and professional support services, as well as build a theoretical model about the Adult Intensive Care Unit environment of care.

**METHOD:**

Grounded Theory, both for the collection and for data analysis. Based on theoretical sampling, we carried out 39 in-depth interviews semi-structured from three different Adult Intensive Care Units.

**RESULTS:**

built up the so-called substantive theory "Sustaining life in the complex environment of care in the Intensive Care Unit". It was bounded by eight categories: "caring and continuously monitoring the patient" and "using appropriate and differentiated technology" (causal conditions); "Providing a suitable environment" and "having relatives with concern" (context); "Mediating facilities and difficulties" (intervenienting conditions); "Organizing the environment and managing the dynamics of the unit" (strategy) and "finding it difficult to accept and deal with death" (consequences).

**CONCLUSION:**

confirmed the thesis that "the care environment in the Intensive Care Unit is a living environment, dynamic and complex that sustains the life of her hospitalized patients".

## Introduction

The care environment involves multiple dimensions of care, and comprises a set of elements that integrate it, and it is necessary to take into account the whole that involves the parts, in the same way as the parts that involve the whole, as is well argued by the idealizer of complex thinking^1^. These aspects, however, are not always considered in the biomedical health care model, whose focus is centered on disease, fragmentation of knowledge, doing and being professional, in which sometimes, not even the being that is cared for is seen as an integrated whole, a being with multiple social relationships, potentiated by the natural and social environment.

In this sense, the care environment requires the creation of conditions favorable to health, promoting a healthy and constructive environment, with harmonious, vitalizing interpersonal relationships that potentiate positive energies for better living^2^.

From this perspective, the health/nursing care environment needs to be better known and understood so that it attains a systemic dimension. It must be apprehended as a circular process that takes into account both the individual who needs care, and the conditions in which this is provided, the human resources and materials available, interpersonal relationships, interactions among health professionals, patients and family members, as well as interactions with the environment.

The care environment in an Intensive Care Unit (ICU), focus of the present study, is an environment intended for the care of seriously ill and unstable patients, who generally remain in the hospital environment, and its complexity is considered high, because it is equipped with leading edge technological and computerized appliances. Here, where aggressive and invasive procedures are performed, the pace is accelerated and the duel between life and death is ever present; and death is frequently imminent^3^^-^ 5.

Therefore, the ICU is frequently stigmatized, and is able to generate erroneous conception with the regard to the care and attitudes of the team^3^. In a similar manner, the ICU is also seen as an environment that generates myths, contradictory sensations and feelings, such as anguish, sadness, pain and suffering, safety and insecurity both in patients and family members, and in professionals who work in this unit.

In this direction, we point out the concepts of order and disorder of Edgar Morin's Theory of Complexity. While the concept of order conveys the ideas of "stability, rigidity, repetition and regularity, in conjunction with the idea of interaction, and necessarily defines itself in its own terms of disorder, which comprises two poles: one objective and the other subjective. The objective pole is that of agitations, dispersions, collisions, irregularities and instabilities, in short, the noise and errors"^6^. Whereas, the subjective pole is that of unpredictability that leads to disorder brings into evidence the uncertainty that brings with it randomness/chance, which is indispensable in the appearance of disorder^1^

Complexity also involves diversity, intertwining and interdependence, and must be understood as an open, broad and flexible system of thought; that is, complex thought. This type of thinking leads to a new comprehension of the world, and to the understanding and acceptance of continuous changes in reality, without denying their multiplicity, randomness and uncertainty, but seeking to coexist with them^7^. Simultaneously, complex thought is also "antagonistic and complementary; contradictory and ambivalent, but is in a constant state of transmutation^8^.

The present study is a summary of the Doctorate Thesis entitled "A sustentação da vida no ambiente complexo de cuidados em Unidade de Terapia Intensiva"^9^^-^ 10, which was based on the following *question of the research*: What is the meaning of the care environment in an Adult Intensive Care Unit, experienced by professionals who work in this unit, managers, patients, family members and the professionals of the supporting services?

The objectives of the mentioned thesis were as follows: to understand the meaning of the care environment in an Adult Intensive Care Unit, experienced by professionals who work in this unit, managers, patients, family members and the professionals of the supporting services, in addition to constructing a theoretical model of the care environment in an Adult Intensive Care Unit.

## Method

The method used was the Grounded theory, and the study was conducted based on the principles of theoretical sampling, so that data collection and analysis were performed in alternative sequences and comprised four consecutive stages. In total, the theoretical model was composed of 39 interviews held with 47 differentiated subjects.

Thus, in the first stage, 10 individual interviews were held with a group of participants formed of health professionals, managers and professionals from the supporting services that worked in the Adult ICU of the "Hospital Universitário Professor Polydoro Ernani de São Thiago" of the Federal University of Santa Catarina (UFSC), located in Florianópolis/SC (ICU 1).

In the second stage, 17 interviews were held with two differentiated groups, so that the first group was composed of eight individual and/or collective interviews held with 16 participants, of whom four were patients and 12 family members. Both were living through the experience of ICU at the time of the interview and/or had lived through this experience at previous times. These interviews were held in the same Adult ICU of Florianópolis/SC (ICU 1) mentioned above, as well as in the home of a patient who had been hospitalized in an Adult ICU in the city of Santa Rosa/RS, 16 months after being discharged. In addition, in this second stage, concomitantly with the interviews held with patients and family members, an endeavor was made to hold another nine interviews with health professionals and those from the supporting service of the mentioned Adult ICU of Florianópolis/SC (ICU 1).

Whereas in the third stage, five individual interviews were held with health professionals and managers of the Adult ICU of a private hospital, namely: the "Hospital São Francisco de Assis" in the city of Santa Maria/RS (ICU 2).

In the fourth and last stage, individual interviews were held with health professionals, manager, patient, family members and professionals of the supporting service of an Adult ICU of a philanthropic hospital, namely: the "Hospital Sociedade Portuguesa de Beneficência" in the city of Pelotas/RS (ICU 3).

Therefore, the interviews were held in three Adult ICUs, located in Florianópolis/SC (ICU 1), Santa Maria/RS (ICU 2) and Pelotas/RS (ICU 3), with the purpose of maximizing the variation among the concepts. The studied ICUs varied, as ICU 1belongs to a large public university hospital. ICU 2 belongs to a small private hospital. Whereas, ICU 3 belongs to a large philanthropic hospital.

Furthermore, 50 hours of participant observation were undertaken in the ICU, and general observations in the ICU 2 and 3 before and after the interviews, as well as 55 hours and 30 minutes of participant observation in two ICUS and in a Semi-Intensive Care Unit of the "Hospital *Klinikum Biel*efeld", in Germany, during the Sandwich Doctorate of the main researcher.

The codification process was performed by means of open, axial and selective coding, in distinct but complementary and integrated phases. For data analysis, the analytical mechanism denominated paradigm was used, recommended by Strauss^11^, as a facilitator instrument that involves an organizational scheme that helps to systematically reunite and put the data into order and classify the emergent connections.

In order to comply with the research ethics criteria, the following were taken into consideration: the recommendations of Resolution No. 196/96, of the National Health Council, on researches involving human beings^12^; approval of the Ethics Committee on Research with human beings of the Dean of Research and Extension of the Federal University of Santa Catarina, Process No. 130/09; approval of the Research Ethics Committee of "Sociedade Portuguesa de Beneficência" of Pelotas/RS, Report (un-numbered) of September 8, 2010, in addition to authorization from the respective Boards and Heads of Nursing, of the mentioned ICUs, with the purpose of validating the study proposal and divulging the information.

## Results

It was possible to construct the substantive theory denominated "Sustaining life in the complex care environment of an Intensive Care Unit", which comprises eight that are intimately inter-related and integrated with one another. Thus, by the integration of these eight categories, the theory "Sustaining life in the complex care environment of an Intensive Care Unit", has two categories as *causal condition*, defined as follows: "Caring for and monitoring the patient continuously", and "Using adequate and differentiated technology", which justify the existence of the ICU. As *context*, it also has two categories; that is to say, "Providing a suitable environment", and "Having worried family members". The *intervening conditions* refer to the category "Mediating facilities and difficulties". In its turn, "Organizing the environment and managing the dynamics of the unit" is the category that was defined as *strategy*, and the *consequences* are with respect to the category "Finding difficulties in accepting and dealing with death", which reflect the difficulty shown by the professionals themselves, who work in an ICU.

Sustaining life in the complex care environment of an Intensive Care Unit

"Sustaining life in the complex care environment of an Intensive Care Unit", in addition to being denominated the substantive theory of this study, it is also considered the central category, or central phenomenon, because it present the central theme of the research, relates to the other categories and integrates them into one another. This category comprises the following sub-categories: "Placing value on the lives of patients"; "Recovering the health of patients"; "Characterizing the environment of the ICU", and "Defining the functionality of the ICU environment".

Fighting for life is the goal of the ICU. Therefore, priority is given to the treatments that maintain life and promote the best possible recovery of patients. The ICU professionals seek to take the greatest care and the pay the most careful attention to recovering the health of the patients; they are on the look-out for details that interfere directly with life; are agile, do their utmost; prevent delays in care so that they do not lose patients' lives, because they want patients to improve and leave the ICU well, and frequently increase the resources to maintain life for a longer time. The ICU team feels satisfied with the recovery of patients' health and this arouses their enthusiasm.

Caring for and monitoring the patient continuously

In this study, "Caring for and monitoring the patient continuously" was determined as one of the causal conditions, because it concerns the actions related to the support given to the seriously ill patient, who requires qualified care, with the help of differentiated technology and professionals who are prepared for the work. This category has the following sub-categories: "Considering the conditions of the patients' hospitalization"; "Describing the conditions of patients in the ICU"; "Associating the care environment with nursing/health care"; "The need to consider the patient as a live and human being"; "Interacting with the patients", and "Observing the professionals".

The care of patients in the ICU is related to direct and intensive care, and to permanent monitoring by the professionals. However, care in the ITU requires not only technical care, directed only towards the biological dimension, but full care of patients, treating them as human beings, with respect, affection and dedication, talking to them, consoling them when necessary and at all times seeing what is better for them, making the patients feel well looked-after in all senses, even when they are in a coma, sedated or unconscious.

Using adequate and differentiated technology

The category "Using adequate and differentiated technology", also considered a causal condition, forms part of the ICU structure; that is to say, it is a determinant condition of the existence of the ICU, and which differentiates it from the other environments. It has the following sub-categories: "Technological Resources" and "Material Resources".

With regard to technological resources, in an ICU, the use of technology that differs from the type used in other care environments is indispensible . An adequate ICU environment involves suitable technology; that is to say technological appliances, such as infusion pumps, respirators, cardiac monitors, oxymeters and others. As regards material resources, in the ICU there is the need for and use of many materials and items of equipment, and they need to be suitable, sufficient and of quality, in order to avoid exposing patients to risks. There is strict control of materials, and generally, there can be no lack of materials here.

Providing a adequate environment

"Providing an adequate environment" refers to the context of the ICU, because it concerns the specific ICU environment that must, as a whole, be presentable, harmonious, pleasant, organized and clean, providing the patients, family members and professionals with well-being. Above all, it must be suitable for the care of patients.

The category "Providing an adequate environment" is composed of the following sub-categories: "Surveying the elements of which the physical space of the ICU are composed"; "Comparing two ICUs based on the construction of a new ICU" and "Requiring an environment for the family members".

The ICU professionals showed great concern with regard to the environment, particularly about the size of the space where patients are found, the presence of natural light, the presence of a window in each box in order to situate patients in time, and to enable them to perceive whether it was day or night; they were concerned about individualizing the patient, preventing one patient from seeing the other; they were alert to the privacy of patients, using curtains around each box.

Having worried family members

The category "Having worried family members" includes the following sub-category: "Reporting the experience of ICU from the family member's point of view". The family members also belong to the ICU context, as they form part of the history of the patients hospitalized in it and are responsible for them, which is the reason why they are worried and have expectations of seeing them improve.

However, the presence of family members in the ICU is very restricted, and they generally com to the ICU only during the patient visiting hours, which are fractionated and generally occur three times a day; that is in the morning, afternoon and at night, totaling around one and a half to two hours of daily visits. One of the studied ICUS had visiting hours only in the afternoon and at night, with only 30 minutes at each time.

Mediating facilities and difficulties

Facilities and difficulties are considered intervening conditions in the ICU environment and are inherent to its existence; that is to say, they also form part of the structural conditions of the unit and need to be mediated and taken into consideration for it to work well. In the ICU, as in other environments, professionals find factors that facilitate care, as well as factors that make it difficult.

The category "Mediating facilities and difficulties" is composed of the following sub-categories: "Professional competence"; "Working conditions"; "Demonstrating concern about professional education and encouragement for training/qualification"; "Needing to integrate theory and practice"; "The presence of stress", and "Dealing with conflicts".

Organizing the environment and managing the dynamics of the unit

"Organizing the environment and managing the dynamics of the unit" was defined as a strategy of the substantive theory "Sustaining life in the complex care environment of the Intensive Care Unit", because it forms part of the process; that is to say, the actions involved in sustaining the life of patients hospitalized in the ICU, seeking to recover their health by means of qualified, humanized and safe care, in the fact of the work of various health professionals, in work that is done as a team, in a continuous manner and with the help of supporting services. It also concerns the care of and improvements in the ICU environment itself, in an endeavor to humanize the environment, when recognizing the presence and importance of the family in the care process, and the transmission of information about the patient to the family members, since the family needs to be kept up to date about the patient's development.

The category "Organizing the environment and managing the dynamics of the unit" is composed of the following sub-categories: "Organizing the environment and optimizing the care"; "Professionals' attributes"; "Depending on the supporting services"; "Working as a team"; "Maintaining the flow of work"; "Aggregating new professionals"; "Transmitting information about the patient to the family"; "Endeavoring the humanize the environment", and "Recognizing the presence and importance of the family in the care process".

Finding difficulties in accepting and dealing with death

The difficulty of professionals in accepting and dealing with the death of patients is considered a consequence of the substantive theory "Sustaining life in the complex care environment in an Intensive Care Unit". This difficulty generates suffering and anguish in professionals in the face of not achieving the desired objective, in addition to fear of the family's reaction, the reasons why they frequently avoid interacting effectively with the family members.

The category "Finding difficulties in accepting and dealing with death" comprises the following sub-categories: "Becoming anguished by the suffering and death of patients"; "Becoming frustrated and sensitive because of the prolonged hospitalization of patients and with the presence of terminal patients"; "Facing death in different ways"; "Avoiding effective interaction with the family members"; "Needing the help of a psychologist for the professionals", and "Needing to approach and work on death with professionals".

## Discussion

It is believed that the Grounded Theory was the most adequate method for this study, due to the relevance to the subjective indicators, and because it enable one to understand the collective significance of the care environment in the Adult ICU, resulting from the process of the research in question, which involved the participant observation and holding in-depth interviews, with the participation of 47 subjects, among them health professionals, managers, intra-hospital support service professionals, family members and patients hospitalized in the ICU, or those who had been discharged from the ICU.

In addition to this, the present study, allied to Edgar Morin's theoretical reference of complexity, from a systemic point of view, provided a view of the set and new understanding with regard to the care environment in an ICU, and the construction of the substantive theory "Sustaining life in the complex care environment of the Intensive Care Unit", which is a useful theory, based on research data, which culminated in the development and integration of eight categories.

Generally speaking, the ICU is considered a specific, critical, specialized area, differentiated from other environments, a great deal of technique and many invasive treatments. It is a complex, closed, organized, extremely dynamic and noisy environment. It has a controlled flow of persons. It is a clean place, with sterile and aseptic procedures. In it severely and critically ill patients are concentrated, who present a high risk of dying and require special assistance and extra care.

According to the Ministry of Health of Brazil, the ICU involves a set of elements grouped in a functional manner, destined to care for severely ill patients or those at risk; that is to say, patients who present some condition that is potentially determinant of their instability, and for this reason they require uninterrupted medical and nursing care, in addition to specialized equipment and human resources^13^.

Recently the International medical literature has reported the effects of ICU design with regard to functionality, safety and well-being of patients and their families. They pointed that the characteristics of ICU design in connection with the needs of patients and their families, are privacy, individual rooms, tranquil environment, exposure to daylight, a view of nature, the prevention of infection, an area for the family and free visiting hours^14^.

The reality of Brazilian ICUs, in general, does not yet contemplate all of these aspects, such as individual rooms for patients, with a view of nature, an area for the family and free visiting hours. However, the country has increasingly invested in improvements in the ICU care environments, making them more welcoming and humanized and extending the visiting times.

A study conducted in Germany, in order to gain better knowledge the situation of families of patients hospitalized in ICUs, has shown that they live with uncertainty, crushing emotions, in addition to having to assume additional responsibilities^15^. According to the authors, the family members at all times place the patient hospitalized in the ICU in first place and always want to be close to him/her, with or without participating in the care. In addition, they seek to obtain honest information, social support, and at all times try to keep on being hopeful about the patient's improvement.

Another study conducted, sought to review the context of the care environment of the ICU with regard to safety of the patients and quality of care, specifically with respect to the association between health and infection and the problems and solutions that involve the interdisciplinary team. The scope of the topics included the actual and future architectural design and trends in *layout*; trends that affect the construction of intensive care units and prevention of construction associated with infections related to care, which involve airborne and waterborne risks and design solutions^16^.

Furthermore, according to these same authors, specific elements include individual occupation, rooms in the intensive care unit with an acuity scale; environmental aspects related tohand hygiene, such as risks related to water, presence and location of the washbasin, management of human resources and residues, selection of surfaces (covering of floor, workbenches, furniture and equipment) cleaning, treatment of antimicrobial agents and similar materials, ultraviolet germicide irradiation, specialized rooms for isolation of patients with airborne infections and protective environments, design of water systems and strategies for the safe use of drinking water.

Another study conducted, compared the design of an ICU in China and Holland based on the established standards. The authors considered that due to the high risk and instability of the patients in ICUs, it is very difficult to design ICUs. However, an ICU project incorporating the *design *of lighting, noise control and other aspects could also improve its management. In addition, they took into consideration, the internal environment in the perspective of planning, analyzed patients and their families, the medical team and the need for space to conduct research into ICU design^17^.

Considering the development of science and social progress, the latter authors increasingly defend the *human-center design*; that is, *design* centered on the human being, from thepoint of view of the users, with the purpose of providing a confortable and efficient ICU environment for the patients, their families and for professionals, satisfying the demands of different requirements, with the development of high technology which, must also be more concentrated one motional balance, and at the sametime, meet the demands of economics, quality and efficiency.

Systemic/complex thinking, in this direction, represents an alternative capable of contributing with rational thought of the Cartesian paradigm, still predominant in the ICUs, in the face of rescuing the human being; by comprehension of the whole, making the connection between the parts and the whole, reason and emotion, objectivity and subjectivity, the environment of the ICU and the other units of intra- and extra-hospital health services, the ICU and the social universe, and the ICU and the environment.

To sum up, the goal of the ICU is to invest in the recovery of patients' health, with the help of differentiated technologies and qualified professionals. Thus, the ICU environment is a place intended for the care of severely ill, unstable and recoverable patients, who are at risk of dying, but who are not hospitalized in the ICU to die.

Nevertheless, due to the severity of the condition in which the patients are found, the limit between life and death becomes a constant presence in this environment, and consequently, the professionals feel satisfaction with the recovery of patients' health, but also feel frustrated and have difficulty in accepting and dealing with their death.

This frustration and difficulty to accept and deal with patients' death in ICU often translates into suffering for professionals. A study that investigated the feelings of suffering of nurses in the ICU, showed that the suffering is related to the care of young critical patient, with the patient's family, teamwork, lack of recognition the work done and the technology at work^18^.

## Conclusion

The present study made it possible to understand the collective meaning of the care environment in an Adult ICU from the life and experiences of health professionals, managers, intra-hospital supporting service professionals, family members and patients hospitalized in an ICU, or who have been discharged from an ICU. This, allied to Edgar Morin's theoretical reference of complexity, and seen from a systemic point of view, provided a view of the set of circumstances and new understanding of the care environment in an ICU, as well as the construction of the substantive theory "Sustaining life in the complex care environment in an Intensive Care Unit", a useful theory based on research data, which culminated in the development and integration of eight categories.

"Sustaining life in the complex care environment in an Intensive Care Unit", in addition to being designated a substantive theory, is also considered the central category, or central phenomenon, because it presents the main theme of the research. It is related to the other categories and integrates them into the others, based on the analytical mechanism denominated paradigm recommended by the method used. It enabled the reunion and systematic ordering of the data, providing the integration of the structural conditions that concerned the causal, contextual and intervening conditions, as the process which refers to the strategies of action and interaction of the professionals involved in care in an ICU, in order to arrive at a better understanding of the dynamics, complexity and nature of the facts in this environment.

It was shown that the ICU is a living and dynamic care environment, in which the sustenance of life occurs, and where the best possible recovery of the patients hospitalized in it is desired. Consequently, the ICU health professionals become frustrated and anguished when they are unable to recover the health of patients and they die. In this sense, the following Thesis is confirmed: "The care environment in an Intensive Care Unit is a living and dynamic and complex environment that sustains the life of the patients hospitalized in it".
